# Safety and efficacy of radiotherapy combined with immunotherapy in limited-stage small cell lung cancer a single-arm meta-analysis and systematic review

**DOI:** 10.1371/journal.pone.0337459

**Published:** 2025-11-20

**Authors:** Li Yu, Xinlin Yu, Cheng Ma, Xialin Zhang, Ran Cui

**Affiliations:** 1 Department of Emergency, First People’s Hospital of Neijiang, Neijiang, Sichuan, China; 2 Department of Oncology, Affiliated Hospital of Chengdu University, Chengdu, Sichuan, China; 3 Department of Oncology, First People’s Hospital of Neijiang, Neijiang, Sichuan, China; 4 Department of Oncology, Affiliated Hospital of Southwest Medical University, Luzhou, Sichuan, China; 5 Department of Respiratory and Critical Care, First People’s Hospital of Neijiang, Neijiang, Sichuan, China; Hokkaido University: Hokkaido Daigaku, JAPAN

## Abstract

**Background:**

Limited-stage small cell lung cancer (LS-SCLC) has a poor prognosis despite being potentially curable with standard concurrent chemoradiotherapy. The success of immune checkpoint inhibitors (ICIs) in extensive-stage SCLC has prompted investigation into combining immunotherapy with radiotherapy for LS-SCLC. This systematic review and single-arm meta-analysis aims to synthesize the evidence on this combined modality, providing pooled estimates of efficacy and safety to inform clinical practice and future trials.

**Methods:**

Following PRISMA guidelines, we systematically searched PubMed, Embase, Cochrane Library, and Web of Science through July 2025 for studies evaluating radiotherapy combined with immunotherapy in patients with LS-SCLC. The primary outcomes analyzed included pooled objective response rate (ORR), median progression-free survival (mPFS), and median overall survival (mOS).

**Results:**

Six studies, encompassing 487 patients, met the inclusion criteria. The pooled analysis demonstrated an ORR of 57.7% (95% CI: 24.9–90.5%), a weighted mPFS of 13.6 months (95% CI: 11.3–15.9 months), and a pooled mOS of 33.7 months (95% CI: 26.7–40.7 months). Grade 3−4 treatment-related adverse events occurred in 42.2% of patients. Subgroup analyses revealed that a concurrent treatment sequence yielded a significantly higher ORR compared to sequential approaches (77.6% vs. 65.2% for immunotherapy followed by radiation vs. 25.8% for radiation followed by immunotherapy). Radiation dose was also identified as a critical determinant of efficacy. Anti-PD-L1 agents showed a numerically higher ORR than anti-PD-1 agents (96.0% vs. 65.0%).

**Conclusion:**

The combination of radiotherapy and immunotherapy is a promising therapeutic strategy for LS-SCLC, demonstrating encouraging efficacy outcomes that appear favorable compared to historical benchmarks for chemoradiotherapy alone. Optimizing treatment sequencing, particularly favoring a concurrent approach, is crucial for maximizing clinical benefit. These findings support further investigation in randomized controlled trials to confirm the value of this combined modality and to identify predictive biomarkers for patient selection.

## Introduction

Small cell lung cancer (SCLC) makes up around 15% of all lung cancer cases. It is marked by fast proliferation, early metastatic spread, and therapeutic resistance, which collectively lead to an unfavorable prognosis [1,2]. Limited-stage SCLC (LS-SCLC), which constitutes 30% of SCLC cases, is potentially curable; however, the median survival remains only 16–24 months, with a 5-year survival rate below 30% [3]. The current standard of concurrent chemoradiotherapy achieves high initial response rates but most patients experience early relapse [4].

The revolutionary influence of immune checkpoint inhibitors (ICIs), particularly atezolizumab and durvalumab, has been evident in the management of extensive-stage SCLC, which have been shown to improve survival when combined with chemotherapy, has generated considerable interest in exploring immunotherapy for LS-SCLC [5]. Radiotherapy and immunotherapy demonstrate compelling synergy: radiation induces immunogenic cell death, enhances neoantigen presentation, and reprograms the tumor microenvironment from immunosuppressive to immunopermissive [6].

However, the optimal integration of radiotherapy with immunotherapy in LS-SCLC remains largely unexplored. Critical knowledge gaps persist regarding the synergistic potential of radiation-immunotherapy combinations, including optimal radiation doses and fractionation schemes when combined with ICIs, sequencing of concurrent versus sequential approaches, and whether radiation field design influences systemic immune responses [7,8]. Published studies exploring radiotherapy-immunotherapy combinations exhibit substantial heterogeneity in radiation protocols and immunotherapy timing. Notably, while previous systematic reviews have examined immunotherapy across all SCLC stages or radiotherapy modifications in isolation, none have specifically synthesized evidence on the combined modality approach in LS-SCLC. Here, we present this systematic review and single-arm meta-analysis specifically examining radiotherapy combined with immunotherapy in LS-SCLC, aiming to provide preliminary pooled estimates of clinical outcomes, identify critical research priorities that may inform ongoing clinical trials in this challenging disease.

## Methods

### Study design and registration

This systematic review and single-arm meta-analysis was conducted according to PRISMA guidelines and registered prospectively in PROSPERO (CRD420251107572). The protocol was finalized prior to the initiation of the literature search.

### Search strategy

We systematically searched PubMed, Embase, Cochrane Library, and Web of Science from inception through July 2025. The search strategy combined MeSH terms and keywords: (“small cell lung cancer” OR “SCLC”) AND (“limited stage” OR “LS-SCLC”) AND (“immunotherapy” OR “immune checkpoint inhibitor” OR “PD-1” OR “PD-L1” OR “CTLA-4”) AND (“radiotherapy” OR “radiation”).

### Study selection

Two investigators independently screened 4,601 identified records. Inclusion criteria were: (1) prospective or retrospective studies evaluating radiotherapy combined with immunotherapy in LS-SCLC; (2) reported efficacy outcomes (ORR, PFS, or OS); (3) minimum 10 patients. Exclusion criteria included: (1) studies with <10 patients were excluded to avoid small-cohort random error compromising pooled analysis reliability; (2) duplicate publications; (3) insufficient outcome data; (4) combination with other experimental agents. Searches were restricted to English language publications. Discrepancies were resolved through consensus with a third reviewer.

### Data extraction and quality assessment

Standardized forms captured study characteristics, patient demographics, treatment regimens, and outcomes. The primary endpoints were ORR (RECIST v1.1), median PFS (mPFS), and median OS. Secondary endpoints included toxicity profiles. Study quality was assessed using the Newcastle-Ottawa Scale for retrospective studies and the Institute of Health Economics checklist for single-arm studies, with scores ≥70% considered high quality.

### Statistical analysis

Pooled proportions for ORR were calculated using random-effects models with Freeman-Tukey double arcsine transformation. Median survival times were pooled using weighted averages based on sample size. Heterogeneity was assessed using I2 statistics (>50% indicating substantial heterogeneity).

Prespecified subgroup analyses examined: (1) treatment sequence (concurrent vs. sequential immunotherapy); (2) radiation dose (≥45 Gy vs. < 45 Gy); (3) ICI type (anti-PD-1 vs. anti-PD-L1); (4) performance status (ECOG 0–1 vs. 2). Sensitivity analyses excluded high-bias studies; publication bias was assessed with funnel plots and Egger’s test.

All analyses were conducted with Stata 14.0 (StataCorp, College Station, TX). Statistical significance was defined as a two-sided P < 0.05.

## Results

### Study selection and characteristics

**Fig 1:**
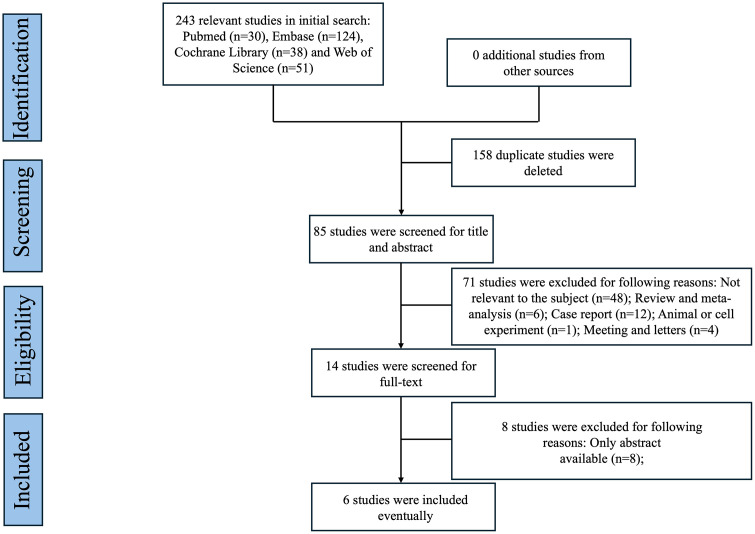
PRISMA diagram illustrating the literature screening and selection procedure.

From 243 records identified through systematic searching, 6 studies [9–14] met inclusion criteria after full-text review, encompassing 487 patients with LS-SCLC treated with combined radiotherapy and immunotherapy. Study characteristics varied in design, sample size and treatment regimens. All studies reported adequate follow-up for survival analyses (median follow-up: 23.1–37.2 months). The study selection process is illustrated in Fig 1, while comprehensive study characteristics are presented in Table 1.

**Table 1:** Characteristics of the studies included in the meta-analysis.

Study	Study type	Sample size	Median follow-up (months)	Intervention	Tumor Stage	Treatment sequence	Radiotherapy dose	Age, years, median (range)	Sex, n		ECOG performance status score, No. (%) a			Smoking status, n (%)			Chemotherapy treatment	Immune checkpoint inhibitors treatment
									Male	Female	0	1	>2	Current	Former	Never-smoker		
Welsh, 2020	Prospective phase I/II study	40	23.1	Pembrolizumab + chemo-radiotherapy	Stage I-IV	Concurrent chemoradiotherapy (etoposide + platinum, 45 Gy in 30 fractions, twice daily) and pembrolizumab (started concurrently, continued for up to 16 cycles). PCI (25 Gy in 10 fractions) at physician’s discretion after treatment.	45 Gy in 30 twice-daily fractions; Prophylactic cranial irradiation: 25 Gy in 10 fractions	64.0 (41.0-79.0)	16	24	13	24	3	11	26	3	Etoposide + platinum	Pembrolizumab
Park, 2022	Prospective phase II study	50	26.6	Durvalumab + chemo-radiotherapy + durvalumab consolidation	Stage I-III	First 2 cycles of chemotherapy (etoposide + cisplatin) plus durvalumab. Non-progressors receive another 2 cycles with concurrent thoracic radiotherapy (52.5 Gy in 25 fractions, once daily). Then durvalumab consolidation (every 4 weeks, max 2 years). Eligible patients receive PCI (25.0 Gy in 10 fractions)	52.5 Gy in 25 once-daily fractions; Prophylactic cranial irradiation: 25.0 Gy in 10 fractions	65.0 (46.0-81.0)	43	7	1	49	0	47		3	Platinum-based chemoradiotherapy	Durvalumab
Cheng, 2024	RCT phase III	264	37.2	Durvalumab or placebo after definitive concurrent platinum-based chemo-radiotherapy	Stage I-III	First standard concurrent platinum-based chemoradiotherapy. Non-progressors receive durvalumab consolidation (every 4 weeks, max 24 months)	60-66 Gy once daily or 45 Gy twice daily	62.0 (28.0-84.0)	178	86	132	132	0	241		23	Cisplatin–etoposide/Carboplatin–etoposide	Durvalumab
Peters, 2022	RCT phase II	78	35	Nivolumab + ipilimumab (experimental) or observation after chemo-radiotherapy + PCI	Stage I-III	First standard chemotherapy (4 cycles platinum + etoposide) plus thoracic radiotherapy and PCI. Non-progressors receive 4 cycles of nivolumab + ipilimumab (every 3 weeks), then nivolumab maintenance (every 2 weeks, max 12 months).	45 Gy in 30 twice-daily fractions (1.5 Gy per fraction) or 56 Gy in 28 once-daily fractions (2 Gy per fraction)	61.1 (37.7-83.2)	50	28	25	50	3	27	51	–	Platinum + etoposide	Nivolumab + ipilimumab
Long, 2025	Retrospective study	29	28.4	ICIs + chemo-radiotherapy	Stage II-III	cCRT (etoposide + platinum + radiotherapy) combined with ICIs (started concurrently, continued for up to 2 years); PCI recommended after cCRT	45 Gy/30 fractions (twice daily) or 60 Gy/30 fractions (once daily)	59.0 (39.0–73.0)	23	6	17	12	0	–	–	–	etoposide + platinum/cisplatin	Not specified in detail (included PD-1 and PD-L1 inhibitors)
Tong, 2024	Retrospective study	26	–	ICIs + chemo-radiotherapy	Stage I-III	Immunotherapy combined with chemoradiotherapy (radiotherapy during chemotherapy, concurrent or sequential)	45 Gy, 1.5 Gy once a day/3 weeks; Prophylactic cranial irradiation (PCI): 25 Gy/2.5 Gy/10 f	64.0 (57.0–68.0)	83	30	25		1	19		7	etoposide + platinum/cisplatin	Not specified in detail (included PD-1 and PD-L1 inhibitors)

PCI, Prophylactic cranial irradiation; RCT, randomized controlled trial; ICIs, immune checkpoint inhibitors; cCRT, concurrent chemoradiotherapy; PD-1, programmed cell death protein 1; PD-L1, programmed death-ligand 1; ECOG, Eastern Cooperative Oncology Group.

### Quality assessment

We assessed the methodological quality of the included studies using standardized tools: the Newcastle-Ottawa Scale (NOS) for the 4 non-randomized studies (evaluating selection, comparability, and outcome assessment across eight criteria) and the Jadad scale for the 2 RCTs (focusing on randomization, blinding, and follow-up). Complete quality assessment results are presented in Table 2.

**Table 2:** Quality assessment of the studies included in the meta-analysis.

NOS for non-randomized studies									
Study	Q1	Q2	Q3	Q4	Q5	Q6	Q7	Q8	TOTAL
Long, 2025	0.5	1	1	1	0.5	0.5	2	1	7.5
Tong, 2024	0.5	1	1	1	0.5	0.5	2	1	7.5
Welsh, 2020	1	1	1	1	1	1	2	1	9
Park, 2022	1	1	1	1	1	1	2	1	9
Modified JADAD Scale for Randomized Controlled Trials									
Cheng, 2024	2	2	2	1					7
Peters, 2022	2	2	2	1					7

NOS, Newcastle–Ottawa Scale.

### NOS for non-randomized studies

The heading signs Q1-Q8 represented: Ⅰ) representative of the exposed cohort; Ⅱ) selection of the non-exposed cohort; Ⅲ) ascertainment of exposure; Ⅳ) outcome present at baseline; Ⅴ) cohort design/analysis; Ⅵ) cohort assessment; Ⅶ) sufficient outcome period; and Ⅷ) follow-up adequacy.

### JADAD scale for reporting randomized controlled trials

Numbers Q1-Q4 in heading signified: Q1: Was the study described as randomized? Q2: Was the method of randomization appropriate? Q3: Was the study described as double-blind? Q4: Was there a description of withdrawals and dropouts?

### Efficacy outcomes

**Fig 2:**
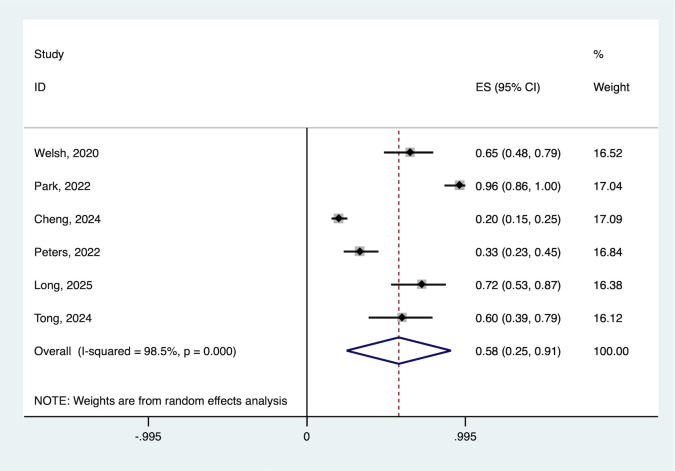
Forest plot of the pooled ORR. ORR, objective response rate.

The pooled ORR across all studies was 57.7% (95% CI: 24.9–90.5%), with moderate heterogeneity (I2 = 98.5%, P < 0.001). Individual study response rates ranged from 20.1% to 96.0%, demonstrating consistent activity of the combination approach. (Fig 2).

**Fig 3:**
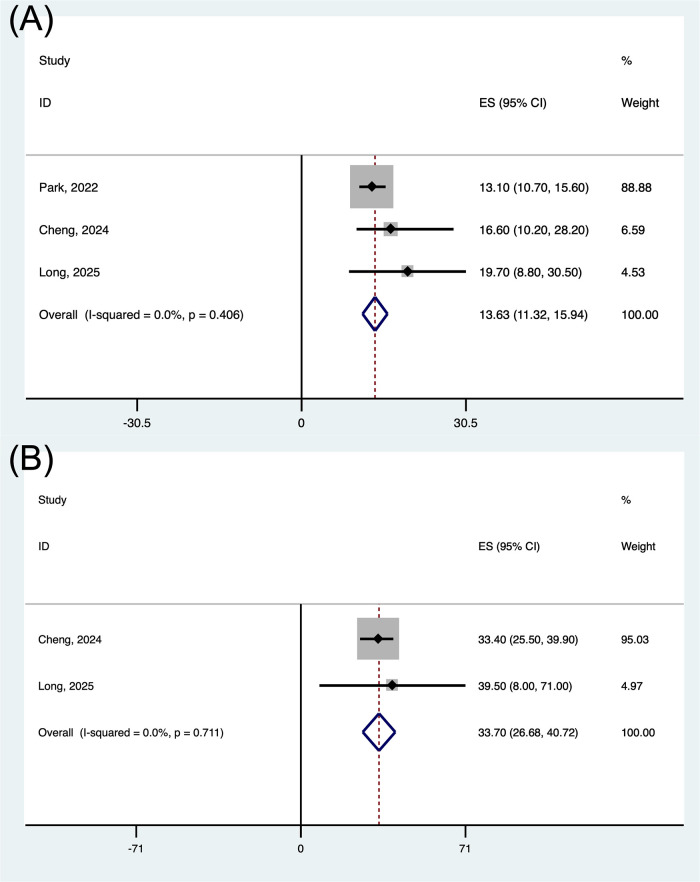
Forest plot of the pooled results for PFS (A) and OS (B). PFS, progression-free survival; OS, overall survival.

For survival endpoints, the weighted mPFS across all studies was 13.6 months (95% CI: 11.3–15.9 months), with individual study estimates ranging from 13.1 to 19.7 months (Fig 3A). The pooled mOS was 33.7 months (95% CI: 26.7–40.7 months) (Fig 3B).

### Safety profile

**Fig 4:**
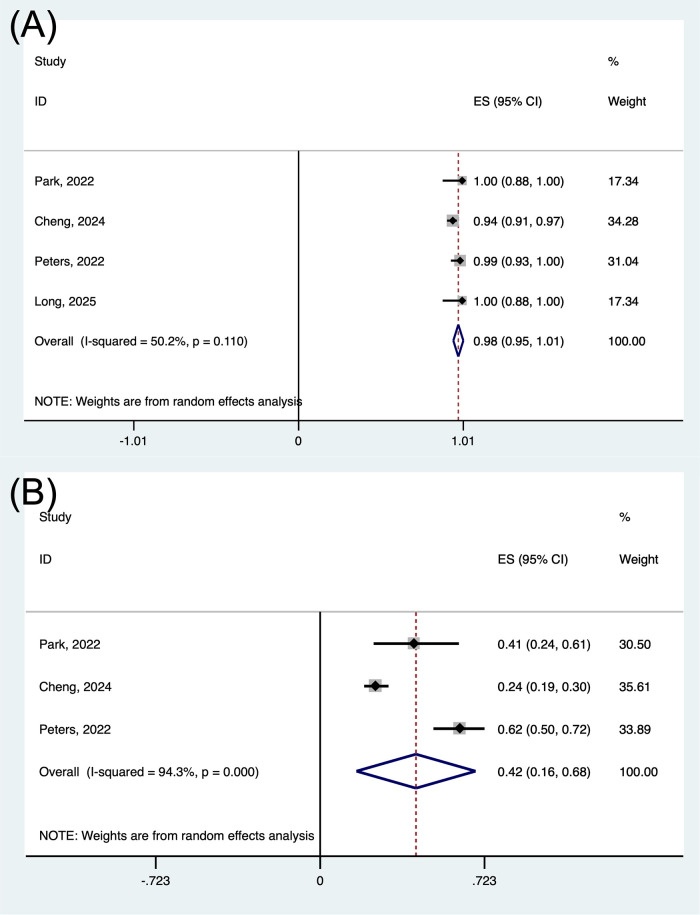
Forest plot of the pooled results for AEs (A) and grade 3-4 AEs (B). AEs, adverse events.

Treatment-related AEs were reported in all studies, with all-grade toxicities occurring in 97.6% of patients (Fig 4A) and grade 3–4 toxicities occurring in 42.2% of patients (Fig 4B). No treatment-related deaths were reported in the included studies. (Table 3).

**Table 3:** Adverse events of the studies included in the meta-analysis.

AEs	All grade	I2,%	≥Grade III	I2,%
	ES,% (95 CI)		ES,% (95 CI)	
Pneumonitis	42.3	98.8	4.4	68.8
Esophagitis	42.5	97.4	NA	NA
Fatigue	40.3	97.5	4.6	68.5
Neutropenia	52.6	16.3	47.2	76.0
Nausea	24.2	95.4	2.6	0.0
Rash	22.3	35.2	1.5	0.0
Pruritus	20.0	15.1	1.5	0.0
Anemia	58.2	81.0	0.7	0.0
Thrombocytopenia	43.9	94.3	12.3	28.1
Cough	19.6	85.3	NA	NA
All	97.6	50.2	42.2	94.3

AEs, adverse events.

### Subgroup analyses

**Fig 5:**
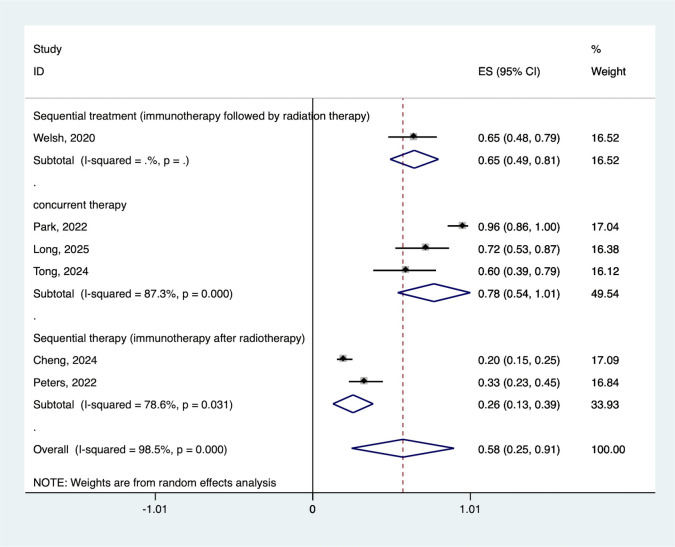
Forest plot of the pooled ORR based on various sequential treatment. ORR, objective response rate.

**Fig 6:**
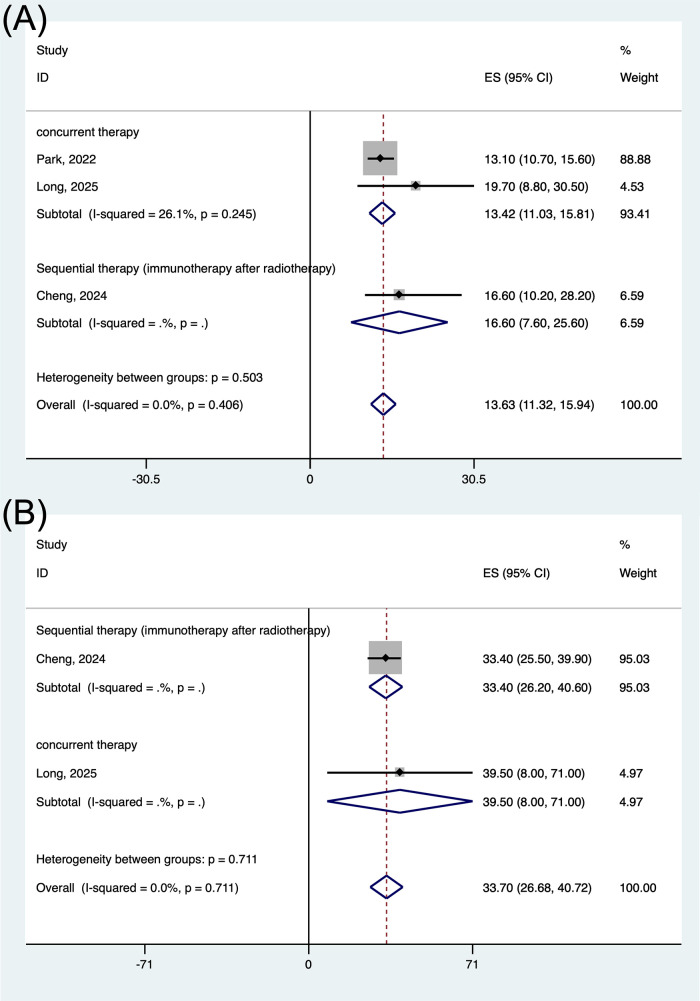
Forest plot of the pooled PFS (A) and OS (B) based on various sequential treatment. PFS, progression-free survival; OS, overall survival.

Treatment sequence significantly influenced outcomes. The ORR of concurrent treatment is better than that of sequential treatment, and among sequential treatments, the ORR of immunotherapy followed by radiation therapy is better than that of immunotherapy after radiotherapy(77.6% vs. 65.2% vs. 25.8) (Fig 5). Prolonged mPFS (13.4 vs. 16.6 months) (Fig 6A) and mOS (39.5 vs. 33.4 months) (Fig 6B) compared to sequential administration.

**Fig 7:**
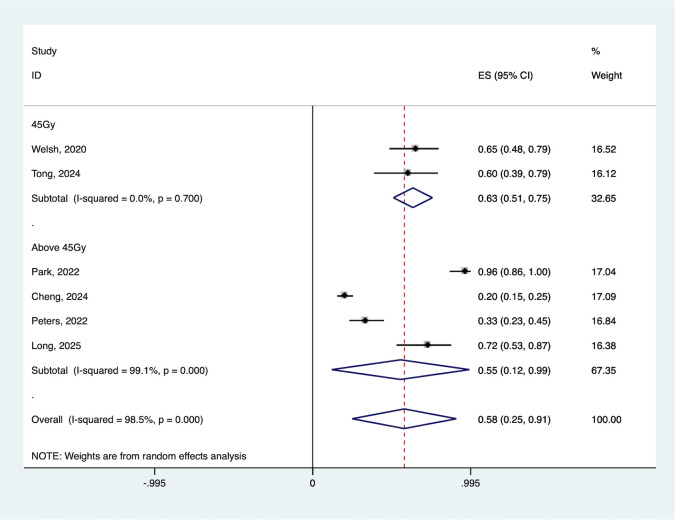
Forest plot of the pooled ORR based on various radiation dose. ORR, objective response rate.

Radiation dose emerged as a critical determinant of efficacy. Patients who received 45 Gy irradiation achieved a better ORR (55.3% vs. 63.1%) than those who received higher doses (Fig 7).

**Fig 8:**
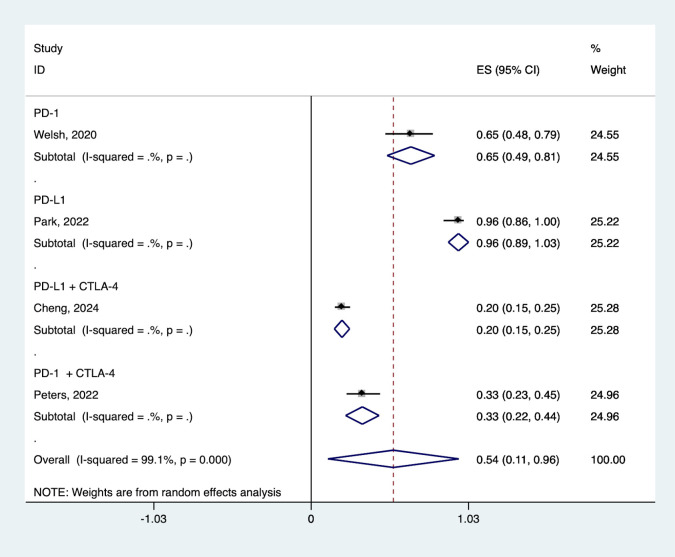
Forest plot of the pooled ORR based on various ICIs. ORR, objective response rate; ICIs, immune checkpoint inhibitors.

ICI selection showed differential effects. Anti-PD- L1 agents demonstrated numerically higher ORR compared to anti-PD-1 agents (96.0% vs. 65.0%,), though this difference was not statistically significant. The combination of two ICIs did not achieve better results, possibly due to immune related adverse events affect treatment efficacy and patient response, resulting in suboptimal ORR in some cases compared to monotherapy ICIs (Fig 8).

**Fig 9:**
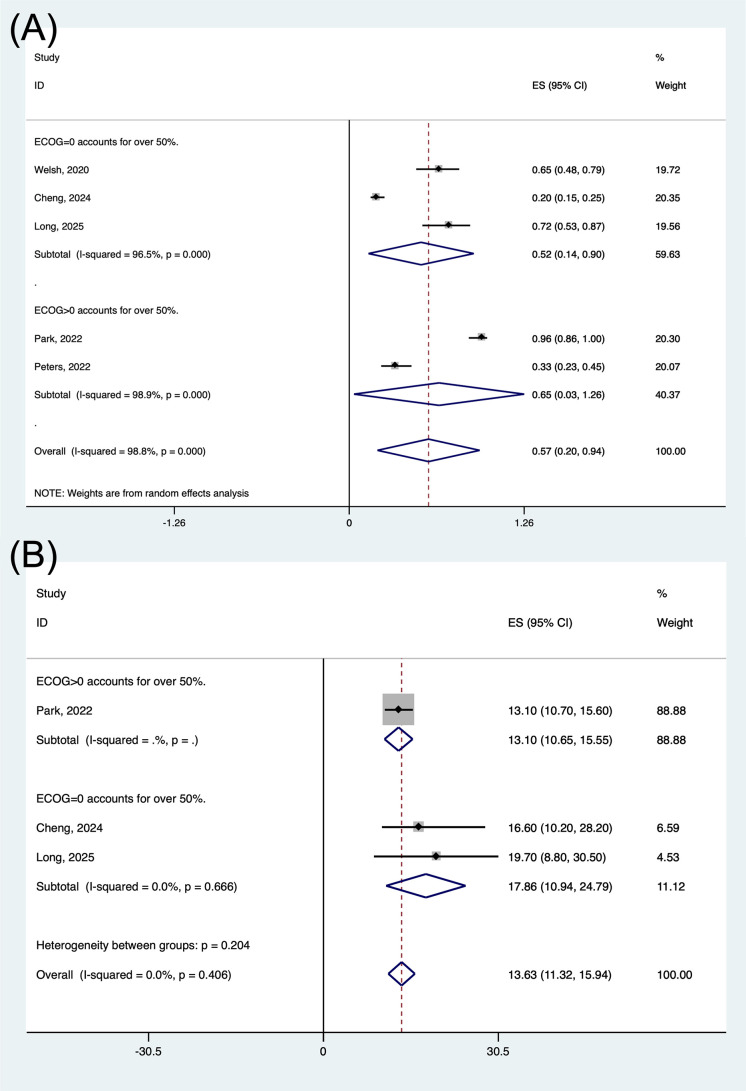
Forest plot of the pooled ORR (A) and PFS (B) based on various ECOG score. ORR, objective response rate; PFS, progression free survival; ECOG, eastern cooperative oncology group.

Compared with more than half of the patients having an ECOG score > 0, more than half of the patients with an ECOG score = 0 did not show better results in all efficacy parameters: ORR (64.8% vs. 51.9%) (Fig 9A), but achieved a longer mPFS (12.8 vs. 7.9 months, P < 0.001) (Fig 9B).

### Sensitivity analysis

A sensitivity analysis was conducted by sequentially excluding one study at a time to assess its impact on aggregated results. Findings showed that pooled results and their 95% confidence intervals remained relatively stable regardless of which study was omitted, confirming the overall reliability of the meta-analysis (S1 Fig).

### Publication bias

To evaluate publication bias and verify the robustness of the meta – analysis results, Egger’s and Begg’s tests were carried out. The results were in line with the main study findings. For ORR (Egger’s P = 0.88, Begg’s P = 0.71), mPFS (Egger’s P = 0.08, Begg’s P = 0.30), mOS (Begg’s P = 1.00) and AEs (Egger’s P = 0.29, Begg’s P = 1.00), no significant publication bias was noted, as stated earlier.

## Discussion

Our systematic review and single-arm meta-analysis provides the first comprehensive evidence synthesis evaluating radiotherapy combined with immunotherapy in LS-SCLC. The pooled analysis of 487 patients from six studies demonstrates efficacy outcomes, with an ORR of 57.7%, median PFS of 13.6 months, and median OS of 33.7 months. These results suggest that incorporating immunotherapy into the treatment paradigm for LS-SCLC may offer meaningful clinical benefits beyond historical benchmarks achieved with chemoradiotherapy alone.

The observed efficacy outcomes are comparable to those reported in historical landmark trials. The CONVERT trial reported median OS of 25–30 months with standard concurrent chemoradiotherapy [15], while our analysis revealed a median OS of 33.7 months with the addition of immunotherapy. Although cross-trial comparisons must be interpreted cautiously, the consistency of improved outcomes across multiple studies suggests a potential benefit. Notably, the ORR of 57.7% aligns with historical response rates of 80–90% for chemoradiotherapy [16], indicating that immunotherapy does not compromise initial tumor response while potentially improving durability of disease control.

Our subgroup analyses reveal critical insights for optimizing treatment delivery. Concurrent treatment yields a higher ORR than sequential treatment. Within the sequential treatment category, immunotherapy followed by radiation therapy achieves a superior ORR compared to radiation therapy followed by immunotherapy, with respective rates of 77.6%, 65.2%, and 25.8%. This finding aligns with preclinical evidence suggesting that radiation-induced immunogenic cell death and neoantigen release are temporally linked processes that may be optimally exploited through concurrent therapy [17]. The dose-response relationship observed with radiation ≥45 Gy supports maintaining adequate radiation doses despite concerns about combined toxicity.

The differential effects observed in ICIs’ selection, with anti-PD-L1 agents showing numerically higher ORR than anti-PD-1 agents (96.0% vs. 65.0%) despite the lack of statistical significance, highlight the need for deeper exploration into their distinct biological behaviors. This numerical advantage might stem from differences in ligand targeting specificity, as PD-L1 inhibitors could potentially block interactions with both PD-1 and CD80, thereby exerting a broader regulatory effect on immune checkpoint pathways [18,19]. The failure of dual ICI combinations to outperform monotherapies, possibly attributed to immune-related adverse events undermining treatment efficacy and patient response, underscores the complexity of balancing therapeutic synergy and safety in combination strategies [20]. Such findings emphasize that optimizing ICI selection—whether choosing between single-agent PD-1 or PD-L1 inhibitors or reconsidering the rationale behind dual-agent combinations—requires careful evaluation of both efficacy signals and tolerability profiles in future clinical investigations.

The contrasting efficacy patterns between patient groups with more than half having ECOG = 0 versus those with more than half having ECOG > 0, where superior ORR did not translate to better outcomes across all parameters but longer mPFS was observed, merit careful interpretation. While the numerical advantage in ORR for the ECOG = 0 group (64.8% vs. 51.9%) suggests some baseline fitness benefit, the statistically significant longer mPFS (12.8 vs. 7.9 months, P < 0.001) highlights that better performance status may particularly influence disease progression dynamics. This discrepancy could reflect that ECOG 0 status, indicating better functional capacity, enables patients to sustain treatment effects over a longer period despite not showing universal superiority in immediate response metrics, emphasizing the multifaceted role of performance status in therapeutic outcomes [21].

Several mechanisms may underlie the observed synergy between radiotherapy and immunotherapy in LS-SCLC. Radiotherapy induces immunogenic cell death, leading to the release of tumor-associated antigens and damage-associated molecular patterns, which in turn activate dendritic cells [22]. Additionally, radiation upregulates MHC class I expression and modulates the tumor microenvironment from immunosuppressive to immunopermissive [23]. The localized nature of LS-SCLC may be particularly amenable to these effects, as the entire tumor burden can be encompassed within the radiation field, potentially maximizing systemic immune activation [24].

The reported grade 3–4 toxicity rate of 42.2% requires careful consideration. This heightened risk necessitates vigilant monitoring and may require protocol modifications, such as stricter lung dose constraints or prophylactic corticosteroid use in selected patients. Future studies should incorporate comprehensive quality-of-life assessments to better characterize the risk-benefit profile.

The landscape of LS-SCLC treatment is rapidly evolving with multiple ongoing trials evaluating different immunotherapy strategies (Table 4). Key questions being addressed include optimal timing (concurrent vs. consolidation), agent selection (PD-1 vs. PD-L1 inhibitors), and combination approaches (dual checkpoint blockade, PARP inhibition).

**Table 4:** Ongoing trials of different immunotherapy strategies for LS-SCLC.

Trial	Agent(s)	Phase	N	Design	Primary Endpoint	Status
NRG-LU005 (NCT03811002)	Atezolizumab	III	544	Concurrent CRT + atezo vs CRT	PFS/OS	Recruiting
KEYLYNK-013 (NCT04624204)	Pembrolizumab + Olaparib	III	672	Concurrent CRT + pembro → pembro + olaparib	PFS, OS	Recruiting
ADRIATIC (NCT03703297)	Durvalumab ± Tremelimumab	III	730	CRT → durva ± treme vs placebo	PFS, OS	Active
ACHILES (NCT03540420)	Atezolizumab	II	212	CRT → atezolizumab	2-year OS	Recruiting
DOLPHIN (NCT04602533)	Durvalumab	II	105	Concurrent CRT + durvalumab	PFS	Recruiting
NCT04189094	Sintilimab	II	140	Induction chemo + sinti → CRT + sinti	PFS	Recruiting

NCT, National Clinical Trials identifier; CRT, chemoradiotherapy; chemo, chemotherapy; PFS, progression-free survival; OS, overall survival; N, sample size.

Biomarker development remains critical for personalized treatment selection. While PD-L1 expression and tumor mutational burden have shown limited predictive value in SCLC, emerging markers including circulating tumor cells [25], inflammatory gene signatures, and specific molecular subtypes may enable patient stratification [26]. Integration of these biomarkers into prospective trials will be essential for optimizing treatment selection [27].

Several limitations warrant acknowledgment. First, the single-arm design prevents definitive conclusions regarding the added benefit of immunotherapy beyond chemoradiotherapy alone. Second, heterogeneity across study designs, immunotherapy agents, and treatment schedules may influence pooled estimates. This is particularly evident in the wide variation of reported overall response rates (ORR) (24.9%–90.5%), with a pooled average of 57.7%. Notably, higher-quality randomized controlled trials (RCTs) reported lower ORRs (15–25% and 23–45%). This discrepancy suggests potential overestimation of immunotherapy efficacy in non-randomized studies due to inherent methodological biases. Selection bias is a significant concern, as non-randomized studies often enroll patients with more favorable baseline characteristics (e.g., better ECOG performance status [0–1], fewer comorbidities, or earlier tumor stage). RCTs mitigate this by using randomization to balance patient characteristics. Outcome assessment bias is also a factor, with non-randomized studies relying on retrospective data or non-standardized radiological evaluation, whereas RCTs typically employ centralized, blinded independent review committees. Differences in follow-up duration may also contribute, as shorter follow-up in some non-randomized studies might inflate short-term ORR. While both study types predominantly used the “chemoradiotherapy followed by immunotherapy” (QT-RT + IT) sequence, this minimizes treatment sequencing as a primary driver of the ORR discrepancy. Third, the relatively short follow-up in some studies may not capture late toxicities or the full spectrum of survival benefits. Finally, it is worth noting a limitation regarding the scope of our findings in relation to our search strategy. While we systematically searched for studies involving CTLA-4 inhibitors, no studies meeting our inclusion criteria utilized a CTLA-4 inhibitor as a monotherapy in combination with radiotherapy. The only included study that involved a CTLA-4 agent employed it in combination with a PD-1 inhibitor, which was analyzed under the ‘combination ICI’ subgroup. Therefore, our results do not provide specific insights into the efficacy or safety of exclusive CTLA-4 and radiotherapy combinations.

## Conclusions

This meta-analysis demonstrates that radiotherapy combined with immunotherapy represents a promising therapeutic strategy in LS-SCLC, with encouraging efficacy outcomes that warrant further investigation in randomized controlled trials. The identification of optimal treatment sequencing, radiation dose thresholds, and predictive biomarkers will be crucial for maximizing clinical benefit while minimizing toxicity. As multiple phase III trials near completion, the treatment landscape for LS-SCLC is poised for transformation, offering hope for improved outcomes in this challenging disease.

## Supporting information

S1 Fig Sensitivity analysis based on (A) ORR, (B) mPFS, (C) mOS, (D) AEs. ORR, objective response rate; mPFS, median progression-free survival; mOS, median overall survival; AEs, adverse events.
(DOCX)

S1 Checklist PRISMA 2020 checklist. (DOCX)

## Abbreviations

LS-SCLC: Limited-Stage Small Cell Lung Cancer
SCLC: Small Cell Lung Cancer
ICIs: Immune Checkpoint Inhibitors
ORR: Objective Response Rate
mPFS: Median Progression-Free Survival
mOS: Median Overall Survival
PD-1: Programmed Cell Death Protein 1
PD-L1: Programmed Death-Ligand 1
CTLA-4: Cytotoxic T-Lymphocyte-Associated Protein 4
AEs: Adverse Events
ECOG: Eastern Cooperative Oncology Group (Performance Status)
Gy: Gray (Radiation Dose Unit)
RECIST: Response Evaluation Criteria in Solid Tumors
NOS: Newcastle–Ottawa Scale
PRISMA: Preferred Reporting Items for Systematic Reviews and Meta-Analyses
PROSPERO: International Prospective Register of Systematic Reviews.
