# Supplementary material for: Safety and efficacy of radiotherapy combined with immunotherapy in limited-stage small cell lung cancer a single-arm meta-analysis and systematic review
Source: PLoS One. 2025 Nov 20;20(11):e0337459. doi: 10.1371/journal.pone.0337459 (PMC12633908; doi:10.1371/journal.pone.0337459)
Supplement: S1 Fig — ORR, objective response rate; mPFS, median progression-free survival; mOS, median overall survival; AEs, adverse events. (DOCX) [file pone.0337459.s001.docx]

# Supporting information


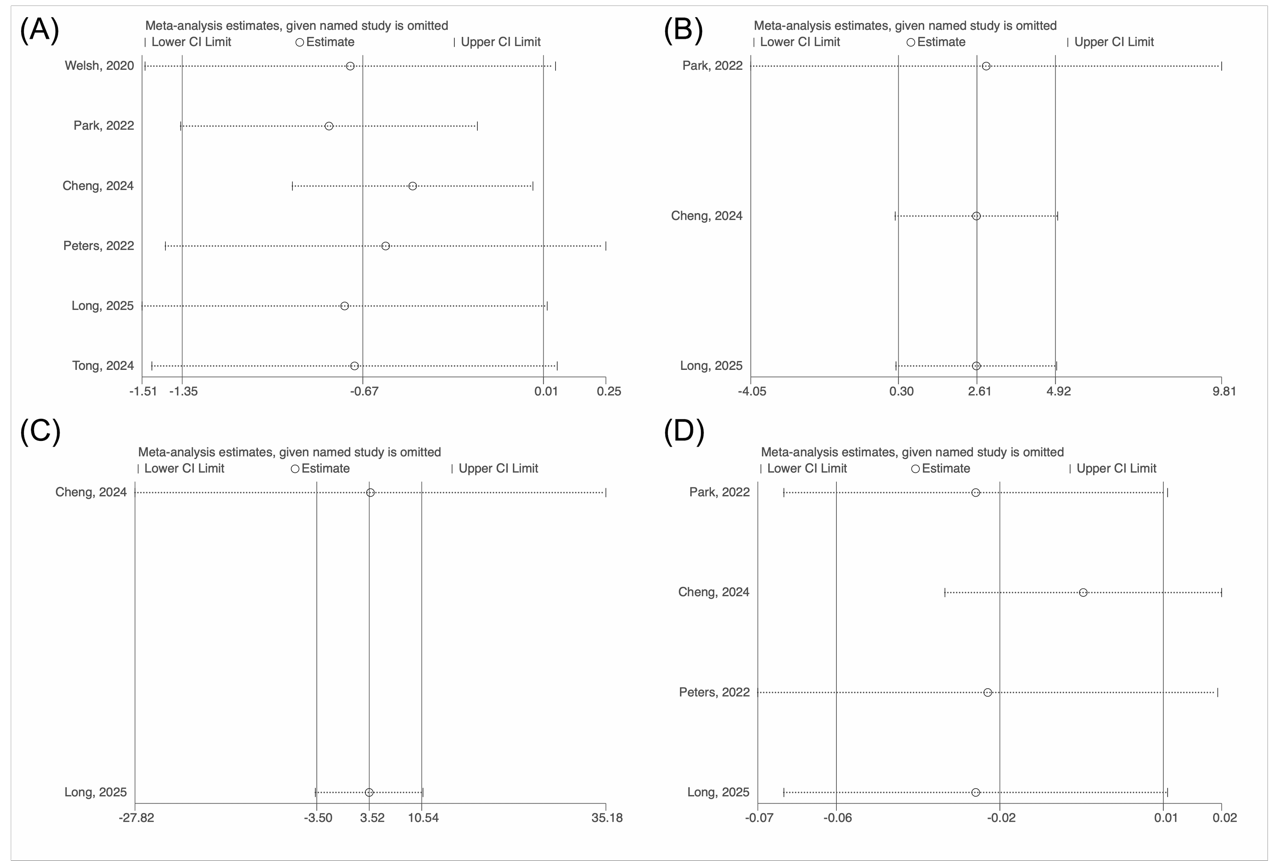


S1 Fig. Sensitivity analysis based on (A) ORR, (B) mPFS, (C) mOS, (D)AEs. ORR, objective response rate; mPFS, median progression-free survival; mOS, median overall survival; AEs, adverse events.
